# Aspirin has a better effect on PIK3CA mutant colorectal cancer cells by PI3K/Akt/Raptor pathway

**DOI:** 10.1186/s10020-020-0139-5

**Published:** 2020-01-30

**Authors:** Zhihang Chen, Chun Wang, Hao Dong, Xing Wang, Feng Gao, Sen Zhang, Xiaolong Zhang

**Affiliations:** 1 Department of Colorectal and Anal Surgery, the First Affiliated Hospital, Guangxi Medical University, Nanning, 530021 China; 2 Department of Gastroenterology, Zhongnan Hospital of Wuhan University, Wuhan, 430000 China

**Keywords:** Colorectal cancer, Aspirin, PI3K/Akt/raptor pathway, PIK3CA mutated

## Abstract

**Background:**

Aspirin, as a non-steroidal anti-inflammatory drug, can improve the survival rate of patients with colorectal cancer, while aspirin is effective in patients with PIK3CA mutant colorectal cancer (CRC). However, the mechanism of aspirin in the treatment of PIK3CA mutated CRC patients remains unclear.

**Methods:**

In this study, immunohistochemistry was used to detect the expression levels of PI3K and Raptor in colorectal cancer patients with PIK3CA mutation and PIK3CA wild-type patients. To demonstrate that aspirin has a better effect on the CRC of PIK3CA mutations in association with the PI3K/Akt/Raptor pathway, we used aspirin to treat PIK3CA mutant CRC cells (HCT-116 and RKO). Subsequently, the CCK8 assay and flow cytometry assay were used to detect the apoptosis of PIK3CA mutant CRC cells before and after aspirin use. Western blot was used to detect the changes of PI3K/Akt/Raptor-associated protein, autophagy protein microtubule associated protein 1 light chain 3 alpha (MAP1LC3A, LC3), beclin 1 (BECN1) and apoptosis protein BCL2-associated X protein/ BCL2 apoptosis regulator (Bax/Bcl2), Caspase 3 after treatment of CRC cells with PIK3CA mutation by aspirin.

**Results:**

Phosphoinositide-3-kinase (PI3K) and regulatory associated protein of MTOR complex 1 (Raptor) protein expression levels were higher in PIK3CA-mutant patients than in IK3CA wild-type patients. The expression of Bax/Bcl2 increased after treatment indicates that aspirin can induce apoptosis of PIK3CA-mutant CRC cells. The expression level of MAP1LC3 (LC3) in cells increases with the concentration of aspirin demonstrates that aspirin can induce autophagy in CRC cells. After 48 h of treatment with aspirin, the phosphorylation of eukaryotic translation initiation factor 4E binding protein 1 (4E-BP1) and ribosomal protein S6 kinase B1 (S6K1) was reduced, cell proliferation has been inhibited. After treatment with aspirin, as phosphorylation of PI3K and Protein kinase B (PKB, Akt) was decreased, Raptor expression was also decreased.

**Conclusion:**

Aspirin can regulate the proliferation, apoptosis and autophagy of CRC cells through the PI3K/Akt/Raptor pathway, affecting PIK3CA-mutant CRC.

## Introduction

Colorectal cancer is a common malignant tumor in the gastrointestinal tract and the prognosis of advanced colorectal cancer is poor. Its pathogenesis is closely related to lifestyle, heredity, and colorectal adenoma (O'Keefe 2016). Early symptoms are not obvious, as the disease progresses, symptoms of blood in the stool and abdominal pain occur, symptoms such as anemia and weight loss will occur in the later stages of the disease (Simon 2016). The disease occurs mostly in middle-aged men, the most common in 40 to 70 years old, the ratio of male to female sex is about 1.5:1 (Siegel et al. 2017). Accumulating evidence indicates that colorectal cancer is a heterogeneous disease, and its therapeutic effect varies from person to person (Yurgelun et al. 2017). This heterogeneity leads to a considerable number of drugs that do not have the desired effect on the treatment of colorectal cancer. So personalized treatment of tumors has become an important research project.

Aspirin (acetylsalicylic acid) is one of the most common non-steroidal anti-inflammatory drugs (NSAIDs). After nearly a hundred years of clinical application, aspirin has proved to have a good effect on alleviating mild or moderate pain (Levesque and Lafont 2000). At the same time, aspirin has an inhibitory effect on platelet aggregation and can prevent thrombosis. It is clinically used to prevent transient ischemic attack, myocardial infarction, artificial heart valve and venous fistula or other postoperative thrombosis (Raber et al. 2019). In recent years, studies have found that long-term use of aspirin can reduce the risk of cancer (Din et al. 2010), and some studies have shown that taking aspirin after surgery can reduce mortality in patients with colorectal cancer (Patrignani and Patrono 2016). In 2016, the US Preventive Services Task Force (USPSTF) published a recommendation for aspirin for primary prevention of CRC, and the efficacy of aspirin in colorectal cancer has been confirmed (Bibbins-Domingo 2016). In 2017, the National Comprehensive Cancer Network (NCCN) noted that aspirin reduces the incidence of CRC in healthy people and reduces the recurrence rate after CRC surgery. However, as a result of individual patient differences, aspirin has different treatment effects for different populations (Cancer and The CGAN 2012). The therapeutic effect on patients with the PIK3CA mutant was significantly better than the PIK3CA wild type (Zumwalt et al. 2017; Liao et al. 2012).

PI3K signaling pathway plays a decisive role in cell growth and proliferation. PI3K signaling becomes abnormal when cancer occurs (Liu et al. 2011). In colorectal cancer, somatic mutations in genes that encode proteins that activate, terminate or transduce PI3K signaling are highly prevalent. The PIK3CA gene encoding catalytic subunit p110α was mutated in about 10% of colorectal cancers (Wang et al. 2018). The two most frequent mutations comprise single amino acid substitutions in two hotspot regions, His1047Arg and Gln545Lys (Samuels et al. 2004). Mutations in PIK3CA will lead to overexpression of phosphorylated PI3K, promoting growth and proliferation of colorectal cancer cells. The mammalian target of rapamycin (mTOR), as a key site for PI3K to link 4E-BP1 and S6K1, plays an important role in the treatment of colorectal cancer (Francipane and Lagasse 2014). And Raptor plays a vital role in the regulation of mTOR (Laplante and Sabatini 2012). Meanwhile, PI3K pathway also plays an important role in autophagy and apoptosis of cells. Whether aspirin affects CRC cells by affecting PI3K/Akt/Raptor, and the relationship between PI3K/Akt/Raptor and PIK3CA mutations needs further validation.

The purpose of this experiment is to explore the reasons why aspirin has a better therapeutic effect on PIK3CA mutant CRC. First, we detected the mutation of PIK3CA gene in CRC patients by gene sequencing. The difference in PI3K between PIK3CA mutant CRC patients and PIK3CA wild type CRC patients was determined by immunohistochemistry, and then the difference in Raptor was determined. We found that the expression of PI3K and Raptor were higher in the CRC with PIK3CA mutation than in the wild type of PIK3CA. Subsequently, we performed an in vitro experiment using aspirin to treat PIK3CA mutant CRC cells. Aspirin was found to promote apoptosis and autophagy of PIK3CA-mutated CRC cells by PI3K/Akt/Raptor pathway, also aspirin was able to inhibit cell proliferation by PI3K/Akt/Raptor pathway. Therefore, it can be concluded that aspirin has a better therapeutic effect on PIK3CA mutation CRC through PI3K/Akt/Raptor pathway.

## Materials and methods

### Antibodies and chemical reagents

Anti-PI3Kinase catalytic subunit alpha antibody (ab135384),Anti-Raptor antibody (ab40768), Anti-S6K1 antibody (ab32359), Anti-S6K1 (phospho T389 + T412) antibody (ab60948), Anti-Bax antibody (ab32503), Anti-Bcl-2 antibody (ab32124), Anti-eIF4EBP1 antibody (ab32024) were purchased from Abcam, USA; PI3 Kinase p110α Antibody(#4255), Phospho-PI3 Kinase p85 (Tyr458)/p55 (Tyr199) Antibody (#4228), Akt Antibody(#9272), Phospho- Akt (Ser473) Antibody (#9271), Phospho-Akt(Thr308) Antibody(#9275), Phospho-4E-BP1 (Ser65) Antibody(#9451), Beclin-1 Antibody(#3738), Caspase-3 Antibody(#9662), LC3 Antibody(#2775) rabbit anti-human monoclonal antibody and Anti-rabbit IgG, AP-linked Antibody(#7054) were purchased from Cell Signaling Technology(CST), USA; Goat anti-Rabbit IgG (H + L) Cross-Adsorbed Secondary Antibody, Alexa Fluor 680 from Thermo Fisher Scientific, China. Aspirin was purchased from Sigma-Aldrich, USA.

### Samples collection

The selection criteria for patients with colorectal cancer above IIIA and IIIA in this study were in accordance with the American Joint Committee on Cancer (AJCC) cancer staging system (eighth edition) issued. Cancer tissues and normal colon tissues were obtained from 104 patients with colorectal cancer stage IIIA and above. The normal colon tissues are taken from more than 10 cm to the edge of the tumor, and it was confirmed no cancer cells infiltrated by pathological examination. All patients did not receive radiotherapy or chemotherapy before, and no other concurrent tumors or diseases. All specimens were taken from the First Affiliated Hospital of Guangxi Medical University. And the Ethics Committee of the First Affiliated Hospital of Guangxi Medical University (Nanning, China) had approved the present study. All patients were tested for mutations in PIK3CA by gene microarray.

### Immunohistochemistry technology

Paraffin sections were inoculated three times in xylene (room temperature, 10 min each), then hydrated in a series of concentration gradients of ethanol (100, 95, 85 and 75% ethanol, room temperature, 10 min each). The sections were placed in 0.01 M citrate buffer (pH 6.0) and boiled (95 °C, 15–20 min) to repair the antigen. Incubate with 0.3% hydrogen peroxide for 30 min at room temperature, then block with goat serum working solution (ZSGB-BIO, Beijing, China) for 15 min at 37 °C. The sections were then incubated with Anti-PI3 Kinase catalytic subunit alpha antibody (1:100) or Anti-Raptor antibody (1:100) for 4 °C overnight. Incubate the second antibody working solution and the third antibody working solution (ZSGB-BIO, Beijing, China) for 30 min at room temperature. After dropping the BAD Chromogenic Kit (ZSGB-BIO, Beijing, China), hematoxylin was used for staining. After installing the coverslip, the microscope was observed. Scoring based on cell number: 0–15% scored 0; 15–30% scored 1; 30–45% scored 2; and > 45% scored 3. Staining intensity was scored as follows: 0 (no staining), 1 (weak staining), 2 (Moderate staining), 3 (Strong staining). The sum of the degree of staining and the number of stained cells is the final criterion. Negative expression score ≤ 3 points, positive expression group score ≥ 4 points.

### Cell culture

Human colon cancer epithelial cells HCT116 cells and RKO cells with PIK3CA c.3140A > G (p.H1047R) mutation were purchased and authenticated from the Shanghai Cell Bank of Chinese Academy of Sciences (Shanghai, China). The cells were cultured in Dulbecco’s Modified Eagle’s medium (DMEM) medium (Gibco, USA), supplemented with 10% fetal bovine serum (Gibco, Grand Island, USA) and 1% penicillin-streptomycin (Solarbio, Beijing, China), at 37 °C in a 5% CO2 humidified atmosphere.

### Cell counting kit-8(CCK-8) assays

The experiment was divided into a blank group containing medium without cells and no aspirin (Ab), a control group containing cells and medium without dosing (Ac), and dosing (2, 4, 6, 8,10,12 mM / L) experimental group (As). HCT-116 cells were plated in 96-well culture plates at a density of 1 × 10^4^ cells per well. After 24 h, different concentrations of aspirin were added, and 4 replicate wells were set in each group. After 12, 24, 48, 72 h of incubation, CCK-8 solution (Dojindo, Japan) was added at 10 μl per well. The absorbance at 450 nm was measured after 4 h of treatment. Calculate the cytotoxicity formula of aspirin on HCT-116 cells as [(Ac-As)/(Ac-Ab)] *100%.

### JC-1 assays

HCT-116 cells in the logarithmic growth phase were seeded in 6-well plates. After attachment, medium with different concentrations of aspirin was added to the wells and set up three repeat wells for each group. After 48 h, the medium was removed, and then add enough JC-1 working solution (Solarbio, Beijing, China) to cover all cells, and incubate at 37 °C, 5% CO2 incubator for 20 min. After removing the JC-1 working solution, the cells were cleaned with PBS. Then the absorbance was measured. The excitation and emission wavelengths of JC-1 monomer are 515 nm and 529 nm, respectively. The excitation and emission wavelengths of JC-1 polymer are 585 nm and 590 nm, respectively.

### Apoptosis assays

The experimental group was divided into the control group and the dosing (2, 4, 8 mM / L) experimental group. Follow the instructions for the AnnexinV-FITC/PI kit (BD Biosciences, USA). The cells were digested and washed with PBS (Solarbio, Beijing, China). After adding 100 μl of 1 × Binding Buffer to resuspend the cells, 5 μl of AnnexinV-FITC was incubated for 15 min, then added with PI dye for 5 min. Finally, 400 μl of 1 × Binding Buffer was added and the cell suspension was detected by FACS Calibur flow cytometer (BD Biosciences).

### Quantitative real time polymerase chain reaction (qRT-PCR) analysis

Detection of MAP1LC3 (LC3) and BECN1 gene expression by qRT-PCR analysis. Total RNA extraction was performed according to the TaKaRa MiniBEST Universal RNA Extraction Kit (TaKaRa, Japan) instructions. The concentration of total mRNA was measured with a spectrophotometer (Nano Drop 2000; USA) and 3 μg mRNA was used to perform reverse transcription. After addition of PrimeScript™ RT Master Mix (Perfect Real Time) kit (Takara, Japan), the RNA was reverse transcribed into cDNA at 37 °C for 15 min, 85 °C for 5 s, and 4 °C for 15 min. Glyceraldehyde-3-phosphate dehydrogenase (GAPDH) was used as the internal reference and the ABI 7500 PCR system (Applied Biosystems, Foster City, CA, USA) was used to perform the quantitative PCR analysis. The 20 μl system working solution was configured according to the TB Green™ Premix Ex Taq™ II (TaKaRa, Japan) instructions. The reaction conditions were: 95 °C for 30 s (reps: 1); 95 °C for 5 s, 60 °C for 30 s (reps: 40). All groups were performed in triplicate and 2 ^-△△CT^ method was used to calculate the relative expression level. QRT-PCR primer sequences are listed below:

- BECN1 FORWARD 5′-CCTCTTCCCTGGAAACAACTAA-3′
- BECN1 REVERSE 5′-TAGTTAGCACAGTAAGCGTTCA-3′
- LC3 FORWARD 5′-CCTGGACAAGACCAAGTTTTTG-3′
- LC3 REVERSE 5′-GTAGACCATATAGAGGAAGCCG-3′
- GAPDH FORWARD 5′-GCAAATTCCATGGCACCGT-3′
- GAPDH REVERSE 5′-TCGCCCCACTTGATTTTGGAGG-3′

### Western blot

After the cells were treated accordingly, the medium was removed and rinsed with PBS. Add RIPA (Solarbio, Beijing, China) containing PMSF (Solarbio, Beijing, China) and Protein phosphorylase inhibitors (Solarbio, Beijing, China) to cleave on ice for 30 min. After collecting the liquid, the supernatant was centrifuged at 13000RPM at 4 °C for 30 min, and the protein concentration was determined by BCA protein assays (Beyotime, Beijing, China). Lysates were resolved by SDS-PAGE and transferred by electrophoresis to polyvinylidene fluoride membranes (Bio-Rad). After the first antibody (Reference *2.1 Antibodies and Chemical reagents*) was incubated at 4 °C over-night, the second antibody was incubated for 1 h. The fluorescence was scanned by an Odyssey Infrared Imaging System (Lico, USA). ImageJ2x was used to evaluate the relative expression of the proteins.

### Plasmid transfection

CRC cells were placed in 24-well plates at a cell concentration of 1*10^5^/mL. Waiting for 24 h cells to adhere, mCherry-GFP-LC3 plasmid (Genepharma Corporation, Shanghai, China) was transfected into cells using Lipofectamine 2000 (Invitrogen, USA) as recommended by the manufacturer. Drug testing of cells after transfection is completed, and the experiment was divided into a control group without aspirin and an experimental group with aspirin (2, 4, 8 mM/L).

### Statistical analysis

All experiments in this study were performed in triplicate. Experimental data were expressed as mean ± standard deviation (SD) and analyzed using SPSS 22.0 software. The differences between the two groups were performed using a t-test. The differences among the groups were tested by a one-way analysis of variance (ANOVA). The difference was statistically significant when *p* < 0.05.

## Results

### Expression of PI3K (p110α) and raptor in PIK3CA mutant colorectal cancer

**Fig. 1:**
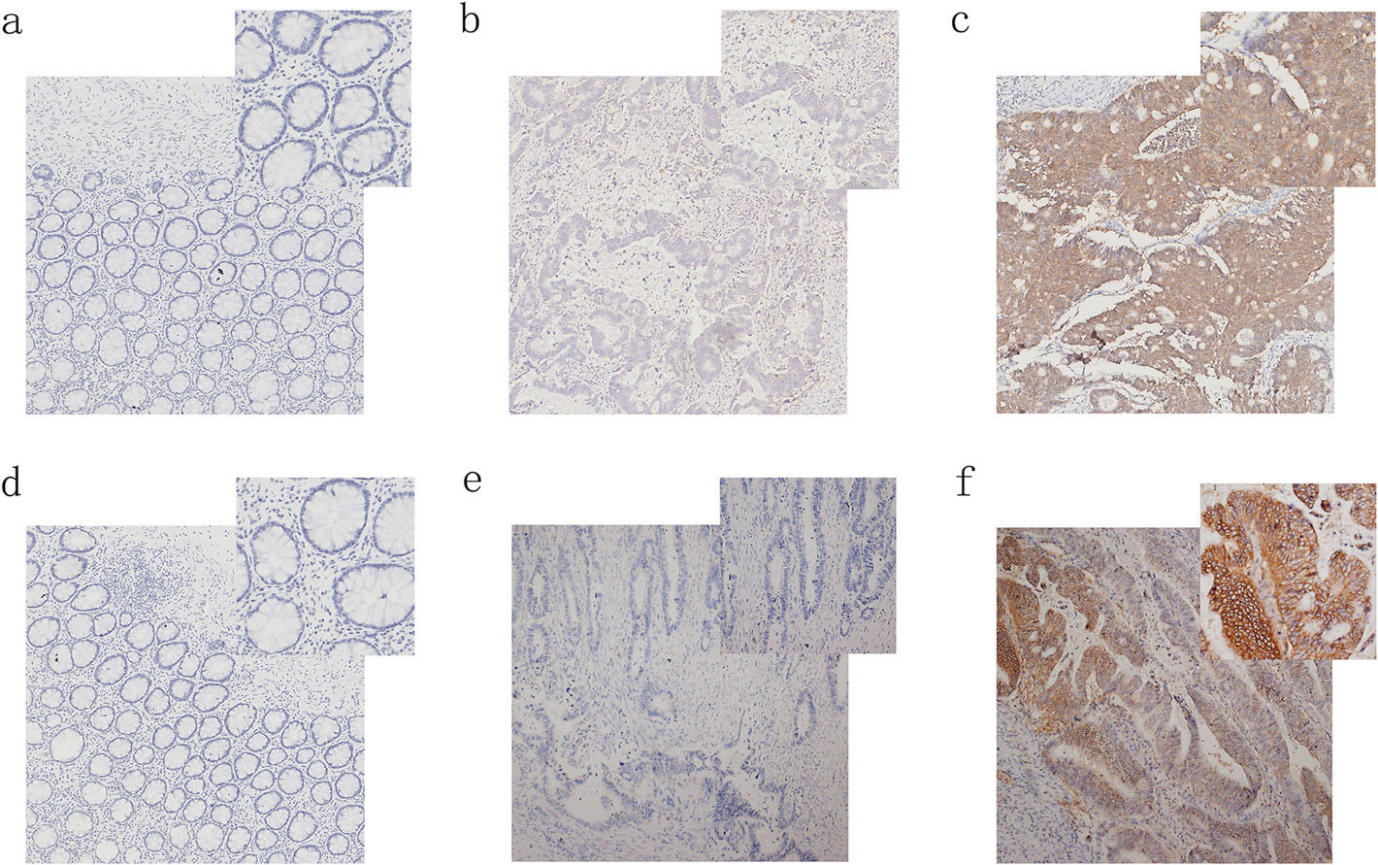
Immunohistochemistry of PI3K-Akt in colorectal cancer tissues and normal intestinal epithelial tissues **a** PI3K (p110α) is negatively expressed in normal intestinal epithelial tissues. **b** PI3K (p110α) negatively expressed in PIK3CA wild-type CRC tissues. **c** PI3K (p110α) is positively expressed in PIK3CA mutant CRC tissues. **d** Raptor is negatively expressed in normal intestinal epithelial tissues. **e** Raptor is negatively expressed in PIK3CA wild-type CRC tissues. **f** Raptor is positively expressed in PIK3CA mutant CRC tissues

We can see from Fig. 1a-c that PI3K (p110α) is extremely low in normal intestinal epithelium of CRC patients, but is mostly deeply stained in cancer tissues and appears in cytoplasm. Combined with immunohistochemistry results of 104 CRC patients, we found that the positive rate of PI3K (p110α) in CRC tissues was 70.2% (73/104). The expression rate of PI3K (p110α) in normal tissues adjacent to cancer was 12.5% (13/104). The expression rate of PI3K (p110α) in CRC tissues is higher than that in normal tissues (*p* < 0.05). Raptor expression mainly occurs in the cytoplasm (Fig. 1d-f). Compared with the expression rate of 5.8% (6/104) in normal tissues, the expression rate of 60.6% (63/104) in cancer tissues was significantly higher (*p* < 0.05). The results are shown in Table 1.

**Table 1:** Expression of PI3K(α110) and Raptor in 104 CRC tissues and expression in 104 normal tissues

Protein	Variables	Negative	Positive	*P*-values
PI3K(α110)	normal tissues	91 (87.5%)	13 (12.5%)	< 0.001
	CRC tissues	31 (29.8%)	73 (70.2%)	
Raptor	normal tissues	98 (94.2%)	6 (5.8%)	< 0.001
	CRC tissues	41 (39.4%)	63 (60.6%)	

The expression of PI3K and Raptor between PIK3CA mutant CRC and PIK3CA wild type CRC can be seen from Tables 2 and 3. The positive expression of PI3K (p110α) in PIK3CA wild-type accounted for 58.2% (39/67). The positive expression in PIK3CA mutant was 91.9% (34/37). The results showed that the expression level of PI3K (p110α) in the PIK3CA mutant CRC tissue was higher than that of the PIK3CA wild type (*p* < 0.05). Raptor is also highly expressed in PIK3CA mutant cancer tissues compared to PIK3CA wild type (*p* < 0.05). The positive expression of Raptor in PIK3CA wild-type accounted for 52.2% (35/67). The positive expression in PIK3CA mutant was 75.7% (28/37). It can also be seen that the positive rate of PI3K (p110α) and Raptor is related to the distant metastasis of tumor (*p* < 0.05), regardless of the gender and age of the patient (*p* > 0.05).

**Table 2:** Clinicopathological variables of 104 CRC patients and their correlation with PI3K (p110α) expression

Variables	All cases (*N* = 104; %)	Negative (*N* = 31; %)	Positive (*N* = 73; %)	*P*-values
Male	67 (64.4%)	18 (26.9%)	49 (73.1%)	0.382
Female	37 (35.6%)	13 (35.1%)	24 (64.9%)	
≤65	66 (63.5%)	21 (31.8%)	45 (68.2%)	0.658
≥65	38 (36.5%)	10 (26.3%)	28 (73.7%)	
M0	73 (70.2%)	27 (37.0%)	46 (63.0%)	0.018
M1	31 (29.8%)	4 (12.9%)	27 (87.1%)	
PIK3CA wild-type	67 (64.4%)	28 (41.8%)	39 (58.2%)	< 0.001
PIK3CA mutant	37 (35.6%)	3 (8.1%)	34 (91.9%)

**Table 3:** Clinicopathological variables of 104 CRC patients and their correlation with Raptor expression

Variables	All cases (*N* = 104; %)	Negative (*N* = 41; %)	Positive (*N* = 63; %)	*P*-values
Male	67 (64.4%)	24 (35.8%)	43 (64.2%)	0.402
Female	37 (35.6%)	17 (45.9%)	20 (54.1%)	
≤65	66 (63.5%)	23 (34.8%)	43 (65.2%)	0.22
≥65	38 (36.5%)	18 (47.4%)	20 (52.6%)	
M0	73 (70.2%)	35 (47.9%)	38 (52.1%)	0.008
M1	31 (29.8%)	6 (19.4%)	25 (80.6%)	
PIK3CAwild-type	67 (64.4%)	32 (47.8%)	35 (52.2%)	0.022
PIK3CA mutant	37 (35.6%)	9 (24.3%)	28 (75.7%)	

### Aspirin can affect PIK3CA-mutant CRC cells by affecting the PI3K/Akt/raptor pathway

**Fig. 2:**
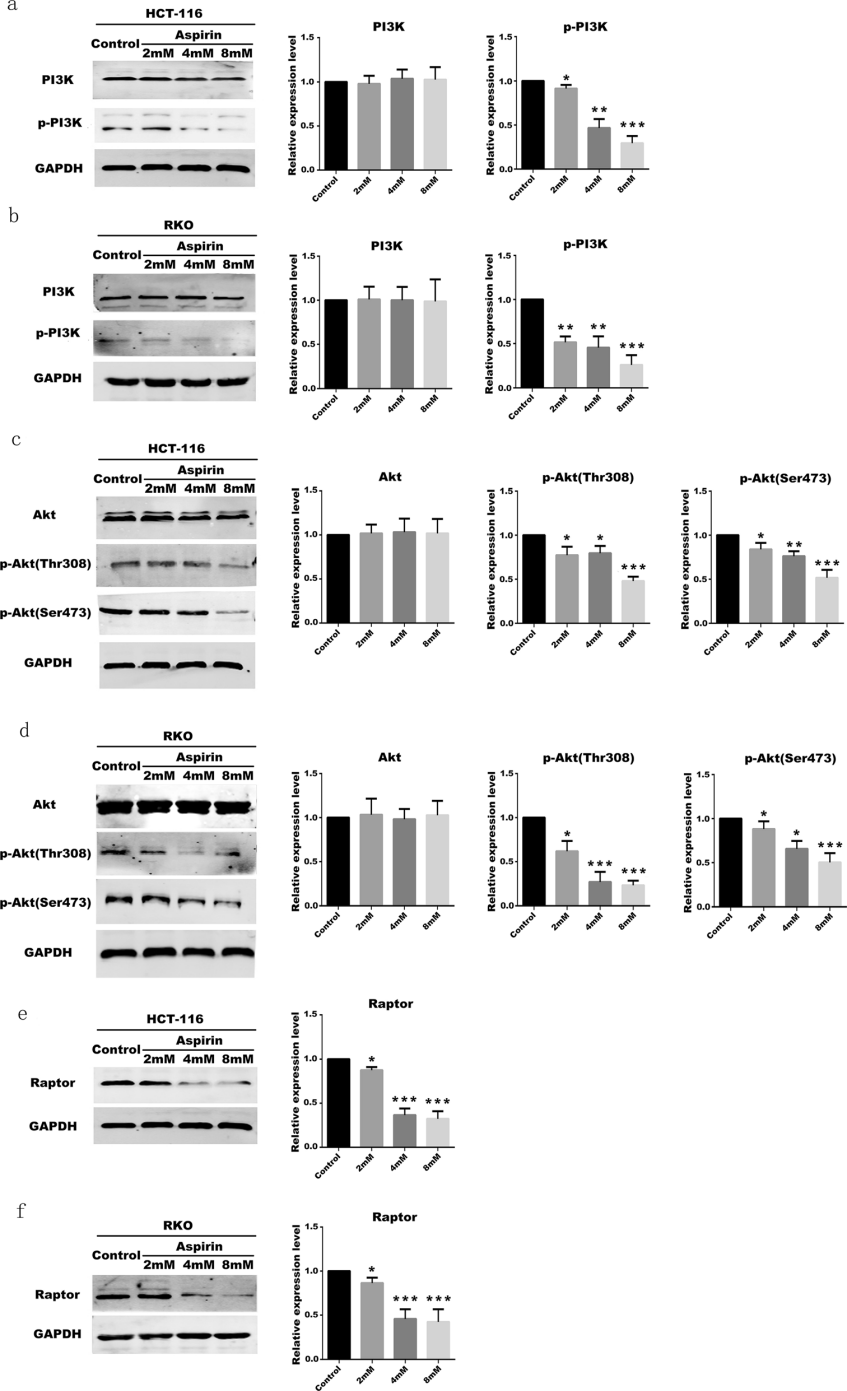
Aspirin affects phosphorylation of PI3K/Akt/Raptor pathway in CRC cells **a** Expression of PI3K protein and protein phosphorylation in HCT-116 cells treated with different concentrations of aspirin. **b** Expression of PI3K protein and protein phosphorylation in RKO cells treated with different concentrations of aspirin. **c** Expression of Akt protein and protein phosphorylation in HCT-116 cells treated with different concentrations of aspirin. **d** Expression of Akt protein and protein phosphorylation in RKO cells treated with different concentrations of aspirin. **e** The results of Raptor protein expression in differently treated groups in HCT-116 cells. **f** The results of Raptor protein expression in differently treated groups in RKO cells. All experiments were repeated 3 times, (*n* = 3) Mean ± SD. *P < 0.05, **P < 0.01 and ***P < 0.001 (vs. normal group)

Members of the IA class in the PI3K family play an important role in cell proliferation and tumorigenesis, whereas PIP3 produced by phosphorylation of PI3K acts as a second messenger to activate PDK1, thereby activating phosphorylation of Akt (Sarbassov et al. 2005). Akt, also known as PKB or Rac, plays an important role in cell survival and apoptosis (Sarbassov et al. 2005). The experiment found that aspirin can affect the activation of PI3K and Akt in PIK3CA-mutant CRC cells (HCT-116, RKO) and inhibit the phosphorylation of PI3K and Akt (Fig. 2a-d). It is apparent from the experimental results that the phosphorylation of PI3K protein in the cells treated with aspirin is decreased. Studies have shown that PI3K can affect the phosphorylation of Akt. After detecting the amount of p-Akt protein in the cells treated with aspirin, it was found that the amount of p-Akt protein was significantly decreased (Fig. 2c, d). mTOR is a bridge between Akt and apoptosis and cell proliferation (Francipane and Lagasse 2014). .Raptor, also known as the egulatory-associated protein of mTOR, is regulated by Akt and affects autophagy and apoptosis (Laplante and Sabatini 2012). .Here, we investigated whether Raptor changes with the use of aspirin in CRC cells with PIK3CA mutations. As a result, the expression of Raptor was lower in the aspirin-treated group than in the control group (without aspirin). And as the concentration of aspirin increases, the expression of Raptor decreases (Fig. 2e, f). Therefore, it can be seen that aspirin can affect PIK3CA-mutant CRC cells through PI3K/Akt/Raptor pathway.

### Effect of aspirin on the apoptosis of PIK3CA-mutant CRC cells

**Fig. 3:**
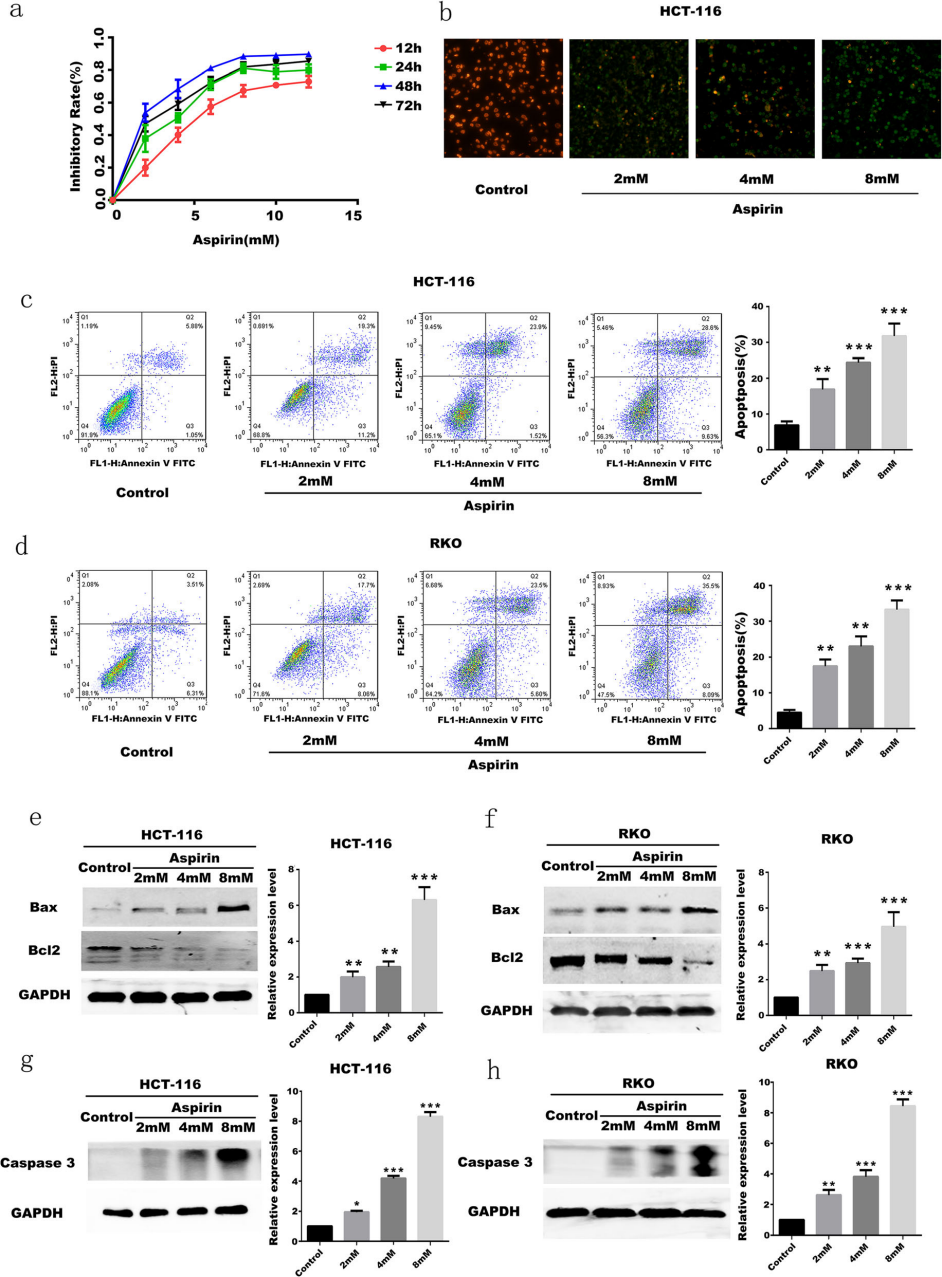
Effect of aspirin on apoptosis of CRC cells **a** CCK8 assay for detection of apoptosis in CRC cells of different treatment times and different concentrations of aspirin. **b** JC-1 Assays were used to determine the effect of aspirin at different concentrations on the mitochondrial transmembrane potential of HCT-116 cells. **c** Flow cytometric analysis for effect of different concentrations of aspirin on apoptosis of HCT-116 cells. **d** Flow cytometric analysis for effect of different concentrations of aspirin on apoptosis of RKO cells. **e** The results of Bax and Bcl2 protein expression in differently treated groups in HCT-116 cells. **f** The results of Bax and Bcl2 protein expression in differently treated groups in RKO cells. **g** The result of Caspase 3 protein expression in differently treated groups in HCT-116 cells. **h** The result of Caspase 3 protein expression in differently treated groups in RKO cells. All experiments were repeated 3 times, (*n* = 3) Mean ± SD. *P < 0.05, **P < 0.01 and ***P < 0.001 (vs. normal group)

To determine if aspirin affects apoptosis in colorectal cancer cells. The effect of aspirin on CRC cells was determined by CCK8 assays. CRC cells HCT-116 with PIK3CA mutation were treated with 0, 1, 2, 4, 6, 8, 10, 12 mM/L aspirin. At the concentration of 8 mM/L, the ability of aspirin to induce apoptosis in HCT-116 cells tends to be stable (Fig. 3a). Meanwhile, the effect of aspirin on HCT-116 cells apoptosis were determined at 12, 24, 48, 72 h. Aspirin induces apoptosis of HCT-116 cells at 48 h to achieve maximum effect (Fig. 3a). At 72 h, the effect of aspirin on promoting apoptosis of CRC cells was diminished, which may be related to the duration of drug effects of aspirin. Therefore, in the follow-up experiments of this paper, the maximum concentration of aspirin was selected as 8 mM/L, and the treatment time was 48 h. The results indicate that the effect of aspirin on apoptosis of colorectal cancer cells is related to the therapeutic concentration and the treatment time (*p* < 0.05). Subsequently, we measured changes in mitochondrial transmembrane potential of HCT-116 cells treated with aspirin at different concentrations. It was found that mitochondrial transmembrane potential decreases with the increase of aspirin dose (Fig. 3b), which further proves that aspirin promotes the apoptosis of HCT-116 cells. And more experiments are needed to verify this conclusion.

In order to verify that aspirin can promote apoptosis of colorectal cancer cells, it was identified by Annexin V-FITC/PI flow cytometric analysis. Two different colorectal cancer cell lines (HCT-116, RKO) with PIK3CA mutation were treated with 0, 2, 4, 8 mM/L aspirin. After apoptosis detection, the apoptosis rate of HCT-116 and RKO cells increased with the increase of aspirin treatment concentration (Fig. 3c, d). According to previous experiments, BAX and BCL2 are identified as apoptosis-related proteins (Koseki et al. 1995). Increased expression of BAX means increased apoptosis of cells (Fukuchi et al. 2001). At the same time, the expression of BCL2 decreases as the apoptosis of the cells increases (Saltzman et al. 1998). 0, 2, 4, 8 mM/L aspirin were used to treat HCT-116 and RKO cells, and then the expression levels of apoptosis-related proteins BAX and BCL2 were detected by Western blot analysis. The results indicate that BAX expression is increased when the concentration of aspirin is increased, and the expression of BCL2 decreases with increasing concentration of aspirin (Fig. 3e, f). Caspase-3, as the main terminal cleaving enzyme in the process of apoptosis, also declined after using aspirin (Fig. 3g, h). It can be seen that aspirin has the effect of promoting apoptosis of PIK3CA-mutant CRC cells. Moreover, the autophagy intensity of CRC cells with PIK3CA mutation increases with the increase of drug concentration.

### Aspirin promotes autophagy in PIK3CA-mutant CRC cells

**Fig. 4:**
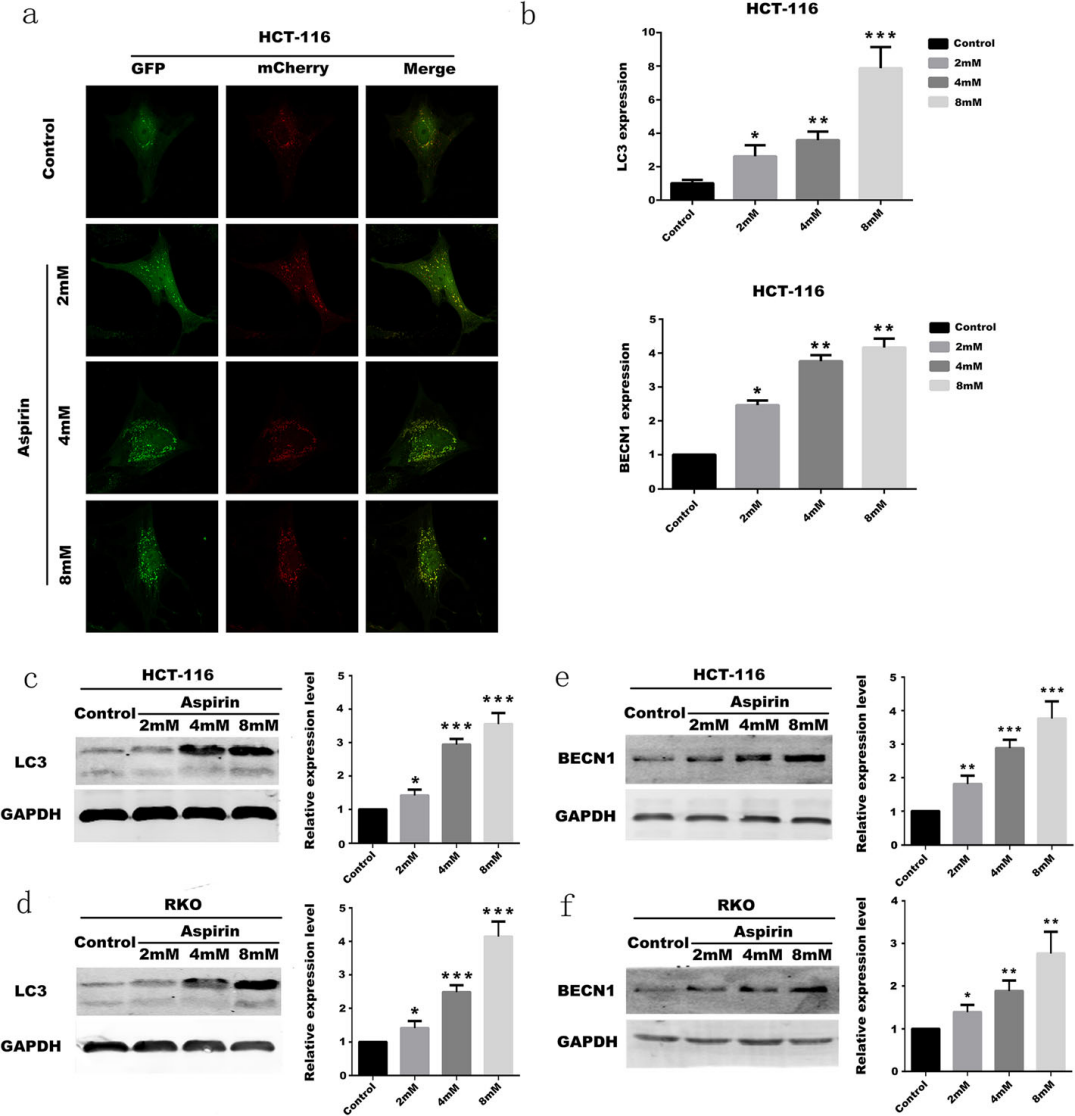
Aspirin induces autophagy in CRC cells **a** mCherry-GFP-LC3 detects changes in autophagic flow in CRC cells treated with aspirin. **b** qRT-PCR was used to detect changes of MAP1LC3 (LC3) and BECN1 in CRC cells after aspirin treatment. (*n* = 3) Mean ± SD. *P < 0.05, **P < 0.01 and ***P < 0.001 (vs. normal group). **c** The results of MAP1LC3 (LC3) protein expression in differently treated groups in HCT-116 cells. **d** The results of MAP1LC3 (LC3) protein expression in differently treated groups in RKO cells. **e** The results of BECN1 protein expression in differently treated groups in HCT-116 cells. **f** The results of BECN1 protein expression in differently treated groups in RKO cells. All experiments were repeated 3 times, (*n* = 3) Mean ± SD. *P < 0.05, **P < 0.01 and ***P < 0.001 (vs. normal group)

Autophagy is a non-apoptotic programmed cell death that participates in the response of cells to external stimuli, participates in developmental processes (Deretic and Levine 2009), and has anti-tumor effects (Mizushima et al. 2008). The study of the effect of aspirin on autophagy in colorectal cancer cells can explore the mechanism of aspirin in the treatment of colorectal cancer. From previous studies, it can be known that MAP1LC3 (LC3) and BECN1 are the key factors in the occurrence of autophagy (Weidberg et al. 2010). When the autophagy of the cells increases, the expression of MAP1LC3 (LC3) is increased (Weidberg et al. 2011),and the expression of BECN1 is also increased. The expression level of MAP1LC3 (LC3) and BECN1 in the cells were determined by qRT-PCR assay. And the PIK3CA-mutant CRC cell (HCT-116) was treated with 2, 4, 8 mM/L aspirin for 48 h. The expression level of MAP1LC3 (LC3) and BECN1 in cells were significantly increased compared with the control group (without aspirin). After the initial test, it can be considered that when HCT-116 cells were treated with aspirin, the expression of MAP1LC3 (LC3) and BECN1 are increased. Moreover, as the drug concentration of aspirin increases, the expression of MAP1LC3 (LC3) and BECN1 are also increased (Fig. 4b). It can be seen that aspirin affects the autophagy of colorectal cancer cells, the relationship between aspirin and autophagy of PIK3CA-mutant CRC cells was further explored.

We confirmed by the results of Q-PCR assays that aspirin can promote autophagy in PIK3CA-mutant CRC cells, and more experiments are needed to prove this conclusion. Different fluorescent signals were observed by transfecting the cytoplasmic mitochondrial mCherry-GFP-LC3 into the HCT-116 cell line. Autophagosomes fuse with lysosomes to form autolysosomes. Due to the acidic environment of autolysosomes, pH-insensitive Cherry fluorescence was primarily observed, while pH-sensitive GFP signals were reduced. In the case of non-autophagy, mCherry-GFP-LC3 is present in the form of diffuse yellow fluorescence, whereas in the case of autophagy, mCherry-GFP-LC3 is present in the form of aggregated yellow spots. And HCT-116 cells were treated with 0, 2, 4, 8 mM/L aspirin. After 48 h, the cells were observed by laser confocal microscopy. It can be clearly observed that as the concentration of aspirin increases, the number of autophagosomes increases (Fig. 4a). Western-blot assays were used to detect the expression of autophagy-associated protein MAP1LC3 (LC3) and BECN1 in HCT-116 and RKO cells treated with different concentrations. It can be seen that compared with the blank group that does not use aspirin, as the concentration of aspirin increases, the expression of MAP1LC3 (LC3) protein is increased (Fig. 4c, d). And the expression of BECN1 protein is also increased (Fig. 4e, f). Through the above experiments, it can be concluded that aspirin can promote autophagy of PIK3CA-mutant CRC cells. And the higher the drug concentration, the stronger the autophagy ability of CRC cells.

### Aspirin inhibits proliferation of PIK3CA-mutant CRC cells

**Fig. 5:**
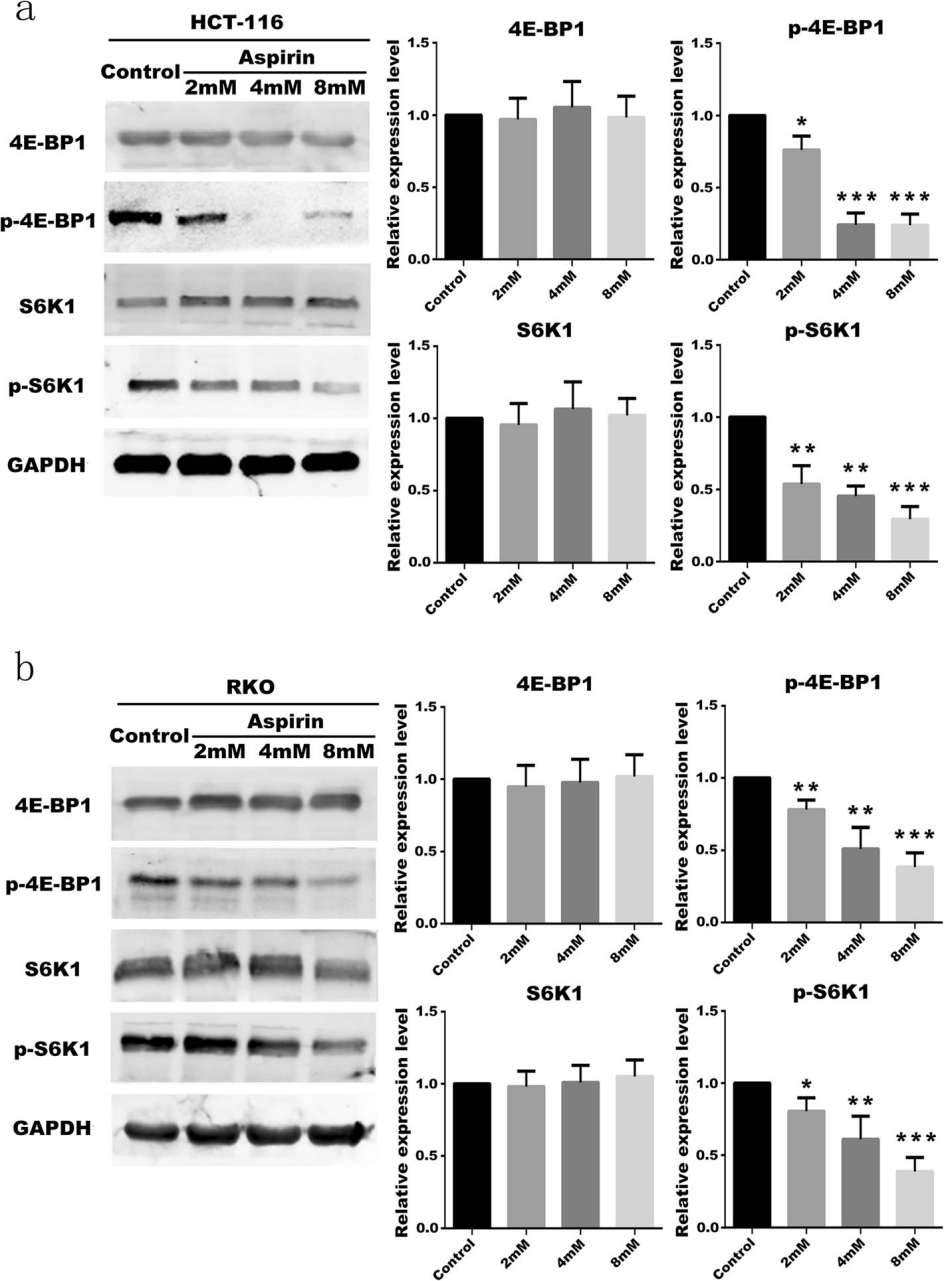
Aspirin inhibits CRC cell proliferation **a** Expression of S6K1, 4E-BP1 protein and protein phosphorylation in HCT-116 cells treated with different concentrations of aspirin. **b** Expression of S6K1, 4E-BP1 protein and protein phosphorylation in RKO cells treated with different concentrations of aspirin. All experiments were repeated 3 times, (*n* = 3) Mean ± SD. *P < 0.05, **P < 0.01 and ***P < 0.001 (vs. normal group)

To investigate whether the proliferation of PIK3CA-mutant CRC cells is affected by aspirin. It has been clarified in the literature that 4E-BP1 and S6K1 proteins are proteins that affect cell proliferation (Fingar et al. 2002). 4E-BP1 and S6K1 are the key factors that influence the translation of cell proteins, and their phosphorylation activation can mediate the nucleotide synthesis and protein synthesis of cells, thus affecting cell growth and proliferation (Ben-Sahra et al. 2013). The phosphorylation of 4E-BP1 and S6K1 proteins in HCT-116 cells and RKO cells without aspirin treatment was determined, and the phosphorylation changes of 4E-BP1 and S6K1 after treatment with aspirin were also obtained. The results showed that phosphorylation of 4E-BP1 and S6K1 was attenuated after aspirin use, and the proliferation of PIK3CA-mutant CRC cells was correspondingly reduced (Fig. 5a, b). When PIK3CA-mutated CRC cells were treated with aspirin, p-4E-BPE and p-S6K1 decreased, indicating that CRC cell proliferation was inhibited.

## Discussion

Although aspirin is primarily used as an antipyretic and analgesic drug, many studies have shown that aspirin can effectively prolong the survival of colorectal cancer patients with PIK3CA mutations (Zumwalt et al. 2017; Domingo et al. 2013). However, the mechanism by which aspirin has a better therapeutic effect on colorectal cancer with PIK3CA mutation is unclear. In this experiment, we investigated the difference in PI3K (p110α) protein expression between PIK3CA mutant and PIK3CA wild-type colorectal cancer. From the results of immunohistochemistry, it was found that the PI3K (p110α) protein expression level in the PIK3CA mutant was significantly higher than that of the PIK3CA wild type. These differences indicate that mutations in PIK3CA result in increased expression of PI3K (p110α). Next we measured the protein expression of Raptor in PIK3CA mutant and PIK3CA wild-type. It was found that the expression of Raptor in the cancer tissue with PIK3CA mutant was higher than the expression of the wild-type PIK3CA colorectal cancer tissue. This indicates that the PI3K/Akt/Raptor pathway is abnormally activated in colorectal cancer with PIK3CA mutation. Therefore, in the following experiments, we focused on whether aspirin has a better effect on PIK3CA mutant colorectal cancer by acting on PI3K/Akt/Raptor pathway.

PI3K/Akt/Raptor pathway plays an important role in the proliferation and migration of cancer cells. PI3K is stimulated by IGF or other signals, the regulatory subunit p85 binds to the tyrosine receptor, thereby attenuating the inhibition of p110α by p85, and then induced the conversion of phosphatidylinositol-4,5-bis-phosphate (PIP2) to phosphatidylinositol-3,4,5-trisphosphate (PIP3) (Mayer and Arteaga 2016). PIP3 acts as a second messenger to activate phosphoinositide-dependent kinase-1 (PDK1), which in turn activates the Akt signal (Mayer and Arteaga 2016). Activated Akt can induce the activation of many downstream factors, and in tumor proliferation, Raptor is one of the most critical factors among these activated factors (Laplante and Sabatini 2012). We performed in vitro experiments to determine phosphorylated protein levels of PI3K and Akt in PIK3CA-mutant CRC cells before and after aspirin treatment. It was found that the phosphorylation process of PI3K and Akt was inhibited by aspirin, and Raptor was also reduced. This experimental result indicates that aspirin can reduce the activation of PI3K and Raptor in CRC cells with PIK3CA mutation.

We further investigated whether aspirin affects apoptosis in CRC cells with PIK3CA mutation. Through in vitro experiments, we learned that aspirin can induce apoptosis of CRC cells, and the apoptosis rate of CRC cells becomes higher as the therapeutic concentration of aspirin increases. Bax and Bcl2 belong to the Bcl2 gene family. Bcl2 is an apoptosis-inhibiting gene (Fukuchi et al. 2001), and Bax not only antagonizes the inhibitory effect of Bcl-2, but also promotes apoptosis (Saltzman et al. 1998). Bax plays a key role in apoptosis induced by mitochondrial stress. The higher the concentration of aspirin used, the greater the number of CRC cells detected during the apoptotic phase. The corresponding Bax/Bcl2 ratio is also increasing. It has been reported that Bax/Bcl2 can be regulated by PI3K signals. PI3K is over-activated in colorectal cancer, whereas activation of PI3K phosphorylates Ser126/Ser112 of Bad in the BCL2 family leading to depolymerization of Bcl2 (Hayakawa et al. 2000). At this time, Bcl2 can play an anti-apoptotic role. When the cells were treated with aspirin, the activation of PI3K was inhibited, and the depolymerization of Bax and Bcl2 was inhibited, and the inhibition of apoptosis of CRC cells was released. It can be concluded that aspirin can promote the apoptosis of PIK3CA mutant CRC cells through PI3K signaling.

PI3K/Akt/Raptor pathway is also closely related to autophagy in cells. Cellular- myelocytomatosis viral oncogene(c-MYC) and Rat sarcoma (RAS), which are downstream transcription factors, are associated with the regulation of autophagy (Shaw and Cantley 2006), while Autophagy related gene 13 (ATG13) is directly involved in the regulation of autophagy (Su et al. 2019). Some studies have confirmed that autophagy has a tumor suppressing function, and autophagy is turned off during tumor formation, which also causes autophagy inhibition of tumor growth to be shut down (Mizushima et al. 2008). At the same time, PI3K-Raptor regulates cell proliferation through 4E-BP1 and S6K1. When the PI3K/Akt/Raptor pathway is activated in PIK3CA-mutant CRC cells, the phosphorylation of 4E-BP1 and S6K1 is increased, and the proliferation of CRC cells is enhanced (Sonenberg and Gingras 1998). The experiment determined that autophagy of PIK3CA-mutant CRC cells in the aspirin-using group was initiated and the autophagy key protein MAP1LC3 (LC3) was up-regulated. At the same time, the CRC cells in the proliferative phase were significantly reduced by the effect of aspirin, and the phosphorylation of 4E-BP1 and S6K1 was inhibited. From the experiment, we can see that aspirin can inhibit the proliferation of PIK3CA mutant CRC cells by PI3K-Raptor signaling. Moreover, aspirin can promote autophagy of PIK3CA mutant CRC cells by PI3K/Akt/Raptor pathway.

As a commonly used and inexpensive drug, aspirin has been accepted by patients and medical institutions, and it has potential chemotherapy effects on colorectal cancer. And aspirin has a more significant therapeutic effect on PIK3CA mutant colorectal cancer. Taken together, our results evaluate the mechanism by which aspirin treats colorectal cancer through PI3K/Akt/Raptor pathway. We studied the expression of PI3K (p110α) and Raptor in colorectal cancer with PIK3CA mutation. The elevation of PI3K (p110α) and Raptor suggests that PI3K/Akt/Raptor pathway has important implications in the treatment of PIK3CA mutant colorectal cancer. The results of in vitro experiments showed that aspirin inhibited the proliferation of PIK3CA-mutant CRC cells through PI3K/Akt/Raptor pathway, induced apoptosis of PIK3CA-mutant CRC cells and enhanced autophagy. Here, it can be preliminarily believed that aspirin can prolong the survival rate of PIK3CA mutant colorectal cancer patients by PI3K/Akt/Raptor pathway. And in vivo experiments will continue.

## Conclusions

Some previous studies have shown that PIK3CA mutant colorectal cancer is resistant to some chemotherapy drugs. And aspirin can affect the signal transduction of PI3K/Akt/Raptor pathway, thereby prolonging the survival rate of PIK3CA mutant colorectal cancer patients. Under the premise of understanding the mechanism of drug action, we can develop more effective chemotherapy programs to improve the survival rate of patients with colorectal cancer.

## Data Availability

The datasets collected and analyzed during the current study are available from the corresponding author upon reasonable request.

## Abbreviations

4E-BP1: Eukaryotic translation initiation factor 4E binding protein 1
AJCC: American Joint Committee on Cancer
Akt: Protein kinase B
ANOVA: A one-way analysis of variance
ATG13: Autophagy related gene 13
Bax: BCL2-associated X protein
Bcl2: BCL2 apoptosis regulator
BECN1: Beclin 1
CCK-8: Cell Counting kit-8
c-MYC: Cellular- myelocytomatosis viral oncogene
CRC: Colorectal Cancer
MAP1LC3 (LC3): Microtubule associated protein 1 light chain 3 alpha
mTOR: Mammalian target of rapamycin
NCCN: National Comprehensive Cancer Network
NSAIDs: Non-steroidal anti-inflammatory drugs
PDK1: Phosphoinositide-dependent kinase 1
PI3K: Phosphoinositide 3 kinase
PIP2: Phosphatidylinositol 4,5 bis phosphate
PIP3: Phosphatidylinositol 3,4,5 trisphosphate
Raptor: Regulatory associated protein of MTOR complex 1
RAS: Rat sarcoma
S6K1: Ribosomal protein S6 kinase B1
SD: Standard deviation
USPSTF: US Preventive Services Task Force
